# Supplementary material for: Difunctional Magnetic Nanoparticles Employed in Immunochromatographic Assay for Rapid and Quantitative Detection of Carcinoembryonic Antigen
Source: Int J Mol Sci. 2023 Aug 8;24(16):12562. doi: 10.3390/ijms241612562 (PMC10454329; doi:10.3390/ijms241612562)
Supplement: Supplementary file 1 [file ijms-24-12562-s001.zip › ijms-2506551-supplementary.pdf]

# Supporting Information

# Difunctional magnetic nanoparticles employed in immunochromatographic assay for rapid and quantitative detection of carcinoembryonic antigen

**Yalin Hu [1,2], Xin Lu [2], Liyue Shen [1,2], Jiahui Dong [1,2], Zhanwei Liang [2], Jie Xie [2], Tao Peng [2,*], Xiaoping Yu [1]
and Xinhua Dai [2,*]**

[1] College of Life Sciences, China Jiliang University, Hangzhou 310018, China; s21090710017@cjlu.edu.cn (Y.H.); p22091055042@cjlu.edu.cn (L.S.); s22090710017@cjlu.edu.cn (J.D.); yxp@cjlu.edu.cn (X.Y.)
[2] Technology Innovation Center of Mass Spectrometry for State Market Regulation, Center for Advanced Measurement Science, National Institute of Metrology, Beijing 100029, China; 13331218159@163.com (X.L.); s20090710033@cjlu.edu.cn (Z.L.); xiejie@nim.ac.cn (J.X.)
* Correspondence: pengtao@nim.ac.cn (T.P.); daixh@nim.ac.cn (X.D.); Tel./Fax: +86-010-64526389 (T.P.)

*Processes of the* **GCNP-ICA** *for CEA detection*

It is similar to the processes of the MNP-ICA development. Firstly, the detection probes for GCNP-ICA were prepared by conjugating CGNPs with mAb against CEA. In detail, 10 μL of 0.2 M $K_2CO_3$ solution was added to 1 mL colloidal gold solution to adjust the pH, then 10 μg of mAbI was added. After incubating for one hour at room temperature, 100 μL of 1% PEG solution and 100 μL of 10% BSA solution were added successively, and stirring for 10 min at room temperature. The mixture was centrifuged at 8000 rpm for 30 min to collect detection probes CGNP-mAb, and the precipitate was resuspended in 100 μL of 0.02 M Tris-HCl buffer (containing 0.5% trehalose, 10% sucrose, 0.5% PVP, 0.1% Tetronic 1307, 0.05% Proclin-300, 1% BSA, 0.1% Tween-20).

The test strips for GCNP-ICA was fabricated as similar as that for MNP-ICA. The mAbII and goat anti-mouse IgG were diluted with PBS buffer (0.01 M, pH 7.4) were sprayed on the NC membrane with 0.8 μL/cm as the T and C lines, and then dried at 37°C for 12-16 hours. The NC membrane, conjugated pad, sample pad and absorption pad were successively pasted on the PVC backing pad by overlapping 2.0 mm. The fabricated test strip was cut into 3.0 mm wide strips, stored at room temperature, and kept dry.

In order to compare the performances of the GCNP-ICA and MNP-ICA under the same conditions, amount of capturing and coating antibodies and other parameters were not optimized systematically. However, the conjugation condition, especially pH, is important for detection probes preparation, pH has been optimized. Under the condition, 3 μL of detection probe was employed, 10 μL of sample and 80 μL of 0.1 M PBS buffer (containing 3% NaCl, 1% Tween-20, 1% BSA) were added onto the sample pad. Detection results can be observed by naked eye for qualitative detection after 15 min, and the colorimetric signals were obtained by the portable immunochromatographic analyzer for quantitative detection.

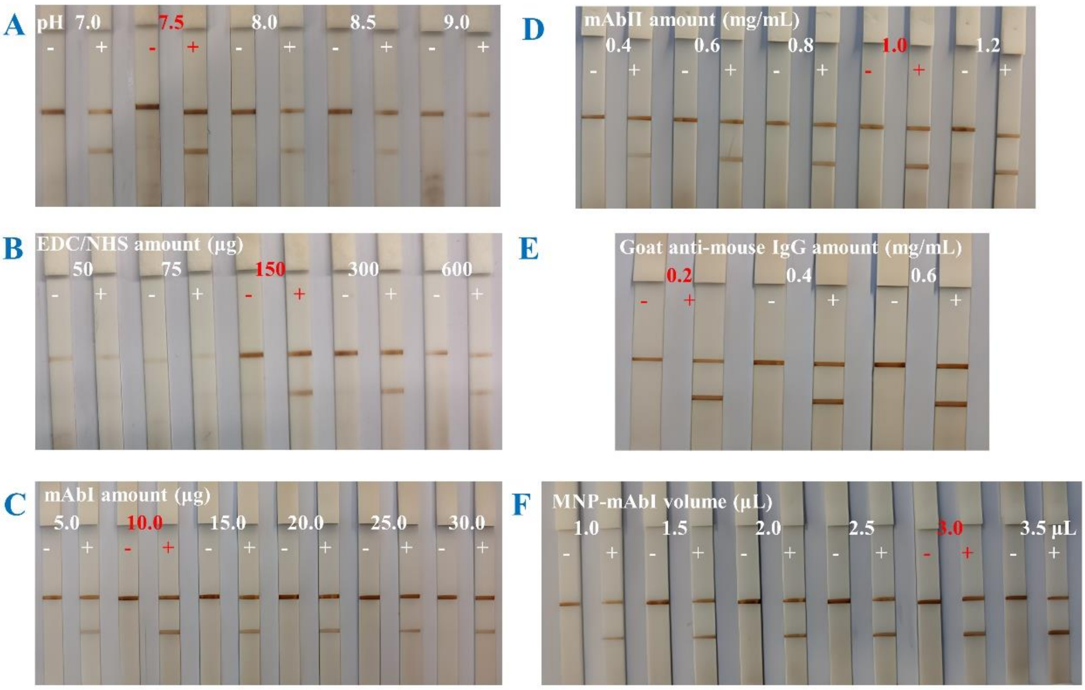


**Figure S1.** Optimal parameters for MNP-ICA development, including pH (A), EDC/NHS amount (B), mAbI amount for labeling (C), amount of mAbII (D) and goat anti-mouse IgG (E) on T and C lines, and volume of MNP-mAbI for each strip (F). ("+" refers to the positive group, that is, the test strip with 50ng/mL CEA serum sample added, and "-" refers to the negative control, that is, the test strip without CEA serum sample.)

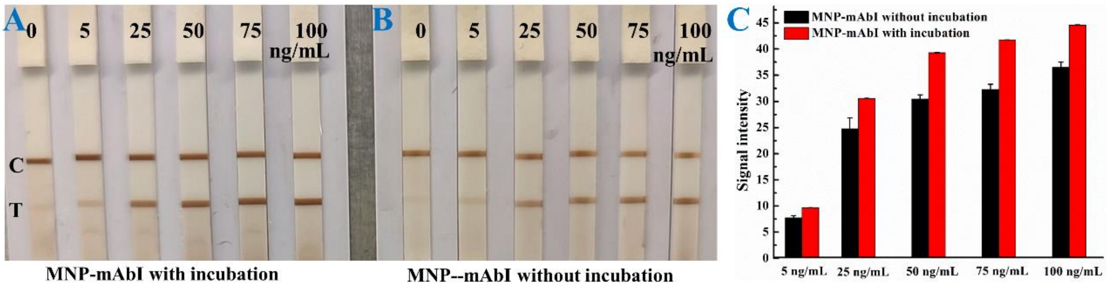


**Figure S2.** Results of MNP-mAbI used with incubation (A) and without incubation (B) before ICA testing, and the signal intensities of T lines obtained and recorded by the portable reader (C).

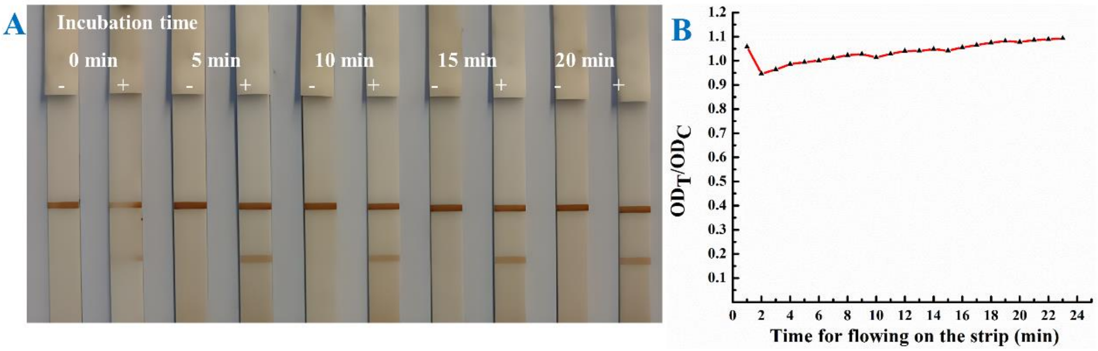


**Figure S3.** Optimization for incubation time (A) and detection time (B) ("+" refers to the positive group, that is, the test strip with 50ng/mL CEA serum sample added, and "-" refers to the negative control, that is, the test strip without CEA serum sample.)

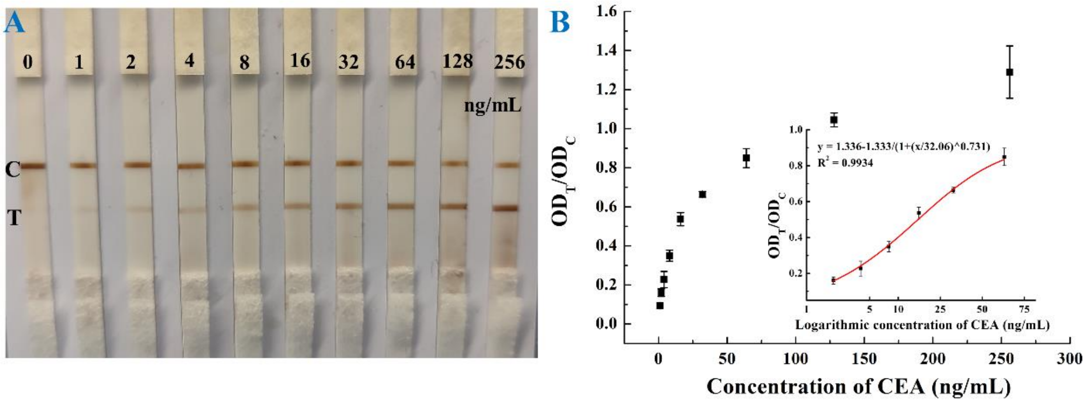


**Figure S4.** Different concentrations of CEA detected by MNP-ICA without incubation. (A) Detection results obtained by naked eye. (B) Calibration curve plotted by CEA concentration *vs.* $OD_T/OD_C$, and the inset is the linear relation between $OD_T/OD_C$ and logarithmic concentration.

A sigmoidal curve fitted by plotting the $OD_T/OD_C$ ratio against the CEA concentration is displayed in Fig.S4B, which indicates the $OD_T/OD_C$ ratio correlated well and with logarithmic concentration from 2.0 ng/mL to 64.0 ng/mL, the regression equation is y = 1.336 - 1.333/(1+(x/32.06)^0.731) ($R^2$ = 0.9934). TheLOD of MNP-ICA without incubation was calculated according to the regression equation and formula: $I_{min} = \overline{X} + 3*\sigma$, where $I_{min}$ is the $OD_T/OD_C$ ratio corresponding to the LOD, $\overline{X}$ is the average ODT/ODC ratio of 11 blank serum samples, σ is the standard deviation. As calculated, the LOD was 0.80 ng/mL, which was about twice higher than that of assay mode with incubation.

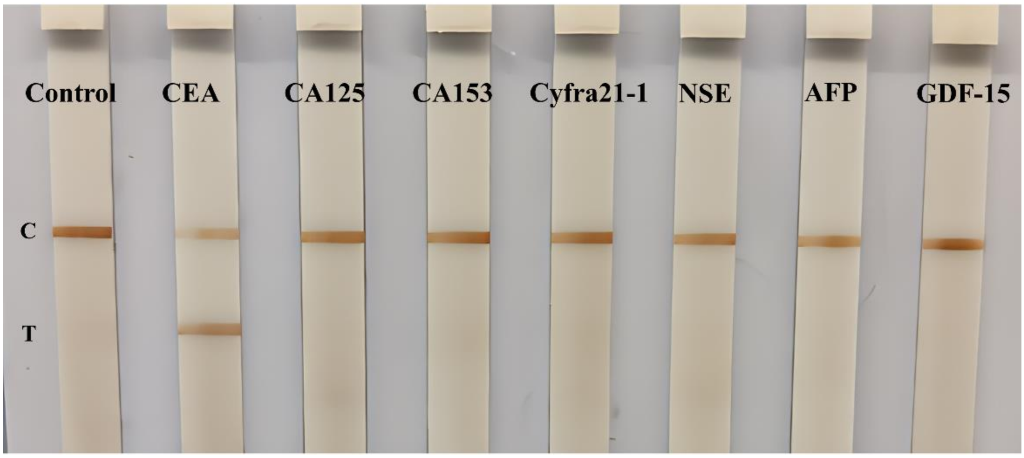


**Figure S5.** Evaluation of the specificity for MNP-ICA with common tumor biomarkers

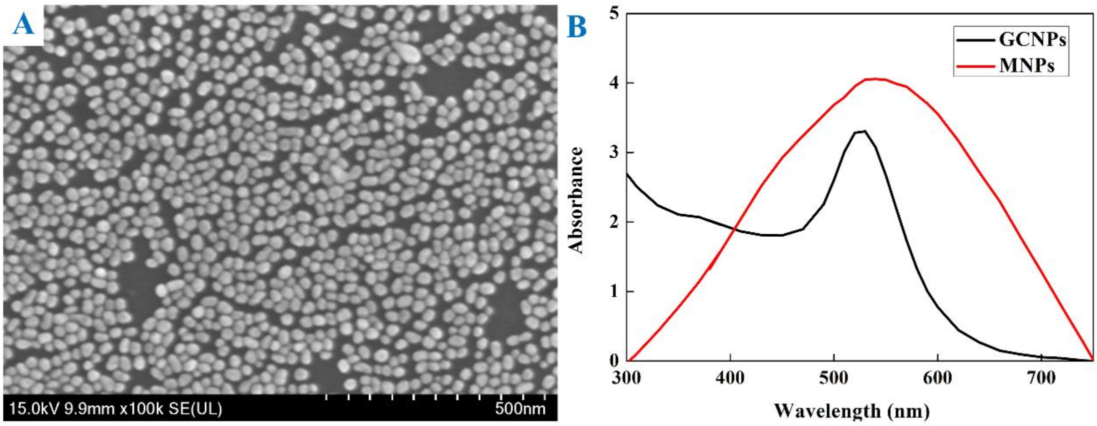


**Figure S6.** SEM image of GCNPs (A) and UV-Vis absorption spectrum for GCNP, MNPs (B)

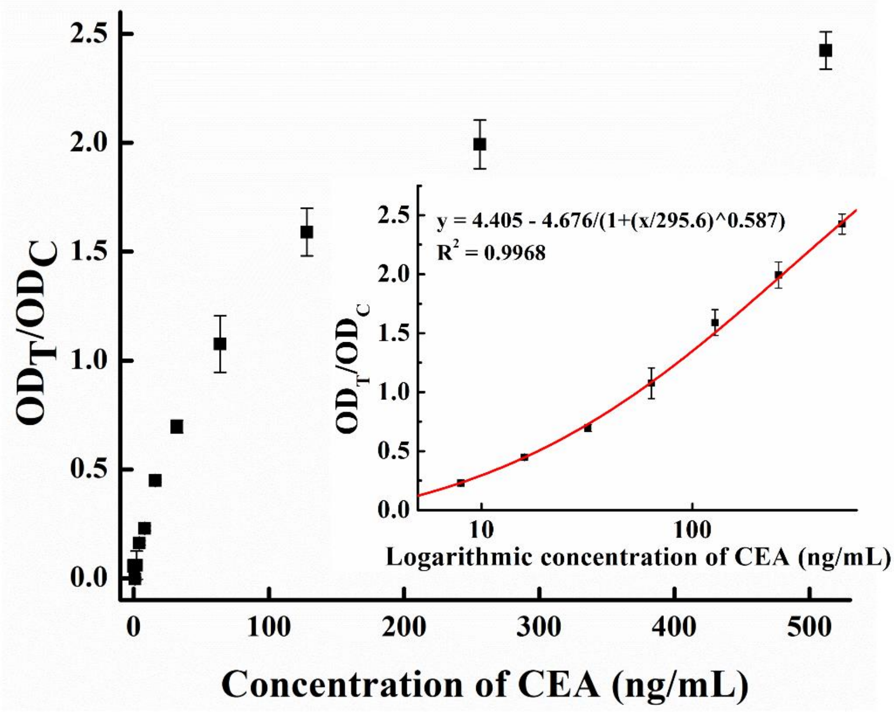


**Figure S7.** Calibration curve of the developed GCNP-ICA for CEA detection, and the inset is the sigmoidal curve fitted with the $OD_T/OD_C$ and logarithmic concentration.

**Table S1.** Relative standard deviation (RSD) of $OD_T/OD_C$ and $OD_T$

| Concentration (ng/mL) | $OD_T/OD_C$ | | $OD_T$ | |
|---|---|---|---|---|
| | Average | RSD(%) | Average | RSD(%) |
| 1.0 | 0.200 | 12.2 | 10.248 | 12.4 |
| 2.0 | 0.260 | 6.6 | 14.250 | 10.3 |
| 4.0 | 0.398 | 10.6 | 20.198 | 18.9 |
| 8.0 | 0.557 | 5.4 | 28.862 | 9.3 |
| 16.0 | 0.719 | 0.2 | 36.448 | 5.2 |
| 32.0 | 0.845 | 4.5 | 42.798 | 3.4 |
| 64.0 | 0.955 | 0.4 | 45.715 | 5.3 |
| 128.0 | 1.076 | 2.8 | 47.294 | 1.9 |
| 256.0 | 2.931 | 4.5 | 48.814 | 4.5 |

**Table S2.** Comparison of ICA methods based on different labels for CEA detection

| Nanomaterials | Linear Range | Time (min) | LOD | Ref. |
|---|---|---|---|---|
| Pdots | / | / | 0.12 ng/mL | Yang et al.[1] |
| Au NPs-CEA-FITC-Ab | 5.0-80 ng/mL | / | 0.1 ng/mL | Wang et al.[2] |
| $NaYF_4$:Yb,Nd@$CaF_2$ | 0.5-10 ng/mL | 8 | 0.5 ng/m L | Han et al.[3] |
| MNPs | 0.25-1000 ng/mL | / | 0.25 ng/mL | Liu et al.[4] |
| MNPs | 1-100 ng/mL | 10 | 0.045 ng/mL | Lu et al.[5] |
| GNT/GNPs | 50-5000 ng/mL | 20 | 15.6 ng/mL | Liu et al.[6] |
| MNPs | 1.0-128 ng/mL | 15 | 0.53 ng/mL | This work |

## References:


[1] Yang, Y. C.; Liu, M. H.; Yang, S. M.; Chan, Y. H. Bimodal Multiplexed Detection of Tumor Markers in Non-Small Cell Lung Cancer with Polymer Dot-Based Immunoassay. *ACS Sens* **2021**, *6* (11), 4255-4264. DOI: 10.1021/acssensors.1c02025.

[2] Wang, J.; Cao, F.; He, S.; Xia, Y.; Liu, X.; Jiang, W.; Yu, Y.; Zhang, H.; Chen, W. FRET on lateral flow test strip to enhance sensitivity for detecting cancer biomarker. *Talanta* **2018**, *176*, 444-449. DOI: 10.1016/j.talanta.2017.07.096.

[3] Han, X. Detection of carcinoembryonic antigen based on $NaYF_4$:Yb,Nd@$CaF_2$ nanocrystals. Harbin University of Technology, 2021.

[4] Liu, F.; Zhang, H.; Wu, Z.; Dong, H.; Zhou, L.; Yang, D.; Ge, Y.; Jia, C.; Liu, H.; Jin, Q.; et al. Highly sensitive and selective lateral flow immunoassay based on magnetic nanoparticles for quantitative detection of carcinoembryonic antigen. *Talanta* **2016**, *161*, 205-210. DOI: 10.1016/j.talanta.2016.08.048.

[5] Lu, W.; Wang, K.; Xiao, K.; Qin, W.; Hou, Y.; Xu, H.; Yan, X.; Chen, Y.; Cui, D.; He, J. Dual Immunomagnetic Nanobeads-Based Lateral Flow Test Strip for Simultaneous Quantitative Detection of Carcinoembryonic Antigen and Neuron Specific Enolase. *Sci Rep* **2017**, *7*, 42414. DOI: 10.1038/srep42414.

[6] Liu Y. Research on the application of carbon-based nanomaterials in immunochromatography analysis. Northwest University, 2018.
